# Androgen receptor and fatty acid oxidation cooperate in ferroptosis evasion in BRAFi resistant melanoma

**DOI:** 10.1038/s41419-026-08578-4

**Published:** 2026-03-23

**Authors:** Marta Redondo-Muñoz, Adria Caballe-Mestres, Julie A. Reisz, Ane Valero-Leria, Ana Olias-Arjona, Paula Aldaz, Angelo D´Alessandro, Claudia Wellbrock, Imanol Arozarena

**Affiliations:** 1 Cancer Signaling Unit, Navarrabiomed, Hospital Universitario de Navarra (HUN), Universidad Pública de Navarra (UPNA), Pamplona, Spain; 2 Health Research Institute of Navarre (IdiSNA), Pamplona, Spain; 3 Institute for Research in Biomedicine (IRB Barcelona), The Barcelona Institute of Science and Technology (BIST), Barcelona, Spain; 4 Department of Biochemistry and Molecular Genetics, University of Colorado Anschutz Medical Campus, Aurora, CO USA; 5 Department of Health Sciences, Universidad Pública de Navarra (UPNA), Pamplona, Spain

**Keywords:** Melanoma, Metabolomics

## Abstract

Melanoma accounts for over 85% of all skin cancer deaths. Current therapies including drugs targeting BRAF and MEK significantly improve the prognosis of metastatic melanoma patients, yet innate or acquired resistance challenges long-term responses. We have shown previously that fatty acid beta-oxidation (FAO) is up-regulated during the acquisition of BRAF-inhibitor (BRAFi) resistance and that the FDA approved drug ranolazine, by targeting FAO attenuates the development of acquired resistance. However, how ranolazine-induced metabolic rewiring increases cell death is unclear. Here we identify ranolazine as a ferroptosis inducer in BRAFi-resistant melanoma, in which FAO serves as a ferroptosis surveillance mechanism. Accordingly, in progressed tumours of BRAFi treated patients up-regulation of FAO regulators correlates with increased expression of ferroptosis markers. BRAFi resistant cells are heavily poised for execution of ferroptosis; they display reduced glutathione levels, higher levels of long-chain polyunsaturated fatty acid (PUFA) membrane-incorporation, and increased membrane-resident phospholipid oxidation, all of which is amplified by ranolazine. Counteracting ranolazine action is MBOAT1/2 mediated phospholipid remodelling, which initiates reduced PUFA membrane-incorporation as ferroptosis surveillance mechanism. We show that the androgen receptor (AR), which is a determinant of BRAFi resistance, controls MBOAT1/2 expression, thereby contributing to ferroptosis resistance. In BRAFi resistant tumours and cell lines, we confirm AR upregulation predominantly in the MITF^low^/AXL^high^ undifferentiated/neural-crest like state, but it also occurs in the MITF^high^/AXL^low^ differentiated melanocytic state. The AR antagonist enzalutamide sensitises AR expressing melanoma cells to RSL3 and erastin independent of phenotype state, but in FAO^high^ BRAFi relapsed tumours AR up-regulation correlates with the undifferentiated/neural-crest like (UD/NC) state, and enzalutamide synergises with ranolazine in ferroptosis-induction in UD/NC cells. Thus, therapeutically combining ranolazine with the AR inhibitor enzalutamide to induce ferroptosis can circumvent dedifferentiation related BRAFi resistance and could increase therapeutic activity and long-term efficacy.

## Introduction

Cutaneous melanoma is a type of skin cancer that originates from melanocytes and is responsible for 80% of skin cancer related deaths. Despite recent developments including targeting the highly deregulated ERK/MAP kinase pathway with overall survival rates of ~44% at 3 and ~28% at 5 years, there is still need for improvement, because most patients eventually relapse due to acquired resistance [1]. One major challenge for targeted therapy is the notorious inter- and intra-tumour heterogeneity of melanomas, based on transcriptionally distinct phenotype cell state [2–5]. These transcriptional states are plastic in nature and their reprogramming enables heterogeneous tumours to adjust to drug exposure during treatment [6–8]. The differentiated melanocytic state (MEL) exerts resistance through MITF [9–13], whereas the neural crest-like (NC) and undifferentiated (UD) states, which express an array of resistance enablers including tyrosine kinases like AXL, not only provide resistance to targeted but also immunotherapy [10, 14, 15].

A metabolic route that is frequently increased in the context of BRAFi resistance is fatty acid beta-oxidation [16–19]. In melanoma FAO and fatty acid transport have also been linked to metastatic dissemination and patient survival [20], and lipid metabolism and FAO are up-regulated in circulating melanoma cells, where this contributes to their improved survival and resistance to ferroptosis [21, 22]. We previously identified increased expression of FAO regulators during the establishment of BRAFi resistance and demonstrated that the addition of the FAO inhibitor ranolazine (RANO) effectively reduces the number of resistant cells [18], but how RANO-induced metabolic rewiring increases cell death is unclear.

BRAFi resistant melanoma cells, particularly of the UD/NC state exhibit low levels of GSH and are therefore sensitive to ferroptosis inducers such as the GPX4 inhibitor RSL3 or the cysteine-glutamate transporter xCT/SLC7A11 inhibitor erastin [5]. Ferroptosis, a cell death triggered by lipid-peroxidation, is primarily induced by the Fe^2+^-dependent peroxidation of membrane-resident long-chain poly-unsaturated fatty acids (PUFAs) enzymatically by lipoxygenases or through nonenzymatic oxidation by free radicals [23, 24]. However, FAO can reduce PUFA levels and thus potentially act as a ferroptosis surveillance mechanism [25]. RANO suppresses FAO regulators in BRAFi resistant cells [18] and considering its potential use in future therapies we wished to fully understand how this translates into reduced survival of melanoma cells in the context of BRAFi resistance.

## Materials and Methods

Detailed methods are described in the Supplemental file

### Cell lines

A375 (female), RPMI7951 (female) and SKMEL28 (male) melanoma cells were from ATCC; M249R cells (female) were a gift from Dr Antoni Ribas [26].

### Single cell RNA sequencing analysis

The generation of single cell RNAseq data from parental A375, resistant A375VR and A375VR_RANO cells using magic (v.2.0.3) [27] as smoothing-based tool has been described [18].

### Metabolomics and lipidomics analyses

Sample preparation, data acquisition and analysis using established protocols [28–30] have been described previously [18].

### Data Analysis and Statistics

Data from GSE80829 [5], GSE50509 [31] and GSE65185 [32] were downloaded from https://www.ncbi.nlm.nih.gov/geo. RSL3 AUC values were from the Cancer Therapeutics Response Portal (CTRP; http://portals.broadinstitute.org/ctrp/) and linked to expression values obtained from https://www.cbioportal.org [33]. GraphPad Prism 10.00 (GraphPad Software, San Diego, CA, USA) was used: one-way ANOVA or two-sided Student’s t test was applied for bar graph analyses, two-way ANOVA for grouped analyses, and Pearson correlation for co-expression analyses. Results are from at least *n* = 3 repeats, and respective *P* values and error bars are reported in the figure legends.

## Results

### BRAFi resistant cells poised to execute ferroptosis display upregulated FAO

**Fig. 1:**
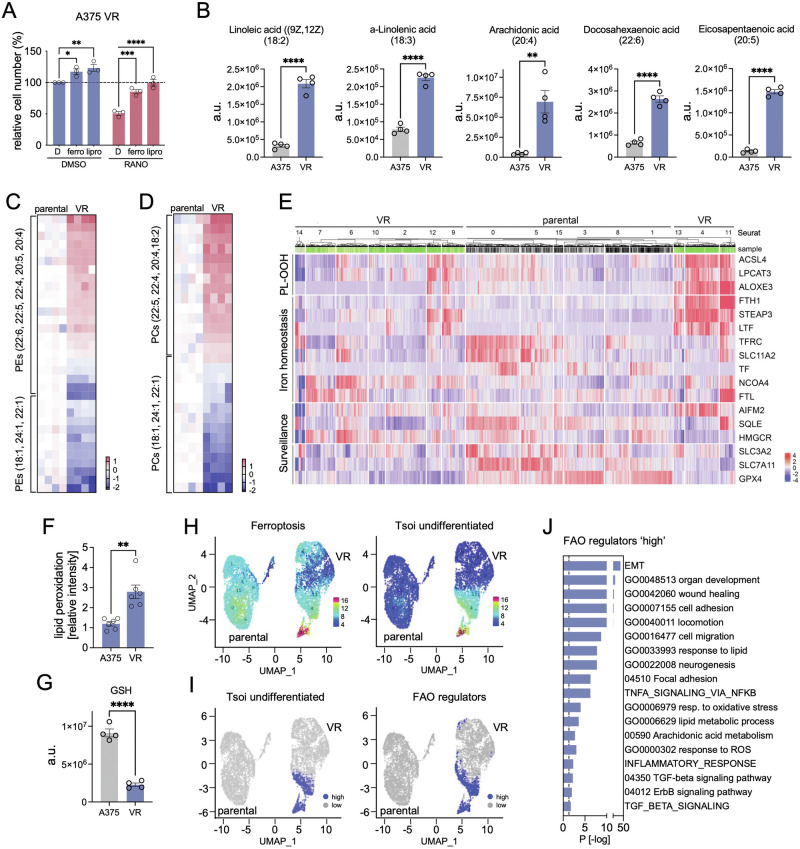
Ferroptosis and FAO are upregulated in BRAFi resistant cells. **A** Colony formation assay (CFA) quantification of A375VR cells treated with DMSO or ranolazine (RANO) in presence of ferrostatin-1 (ferro) or liproxstatin-1 (lipro) or DMSO (D). (*n* = 3, Mean ± SEM, Holm-Sidák test of one-way ANOVA. *p-value ≤ 0.05; **p-value ≤ 0.01; ****p-value ≤ 0.0001). **B** Total levels (integrated peak areas, arbitrary units – a.u.) of indicated fatty acids in A375 parental versus resistant A375VR cells. (*n* = 4, Mean ± SD, two-tailed unpaired *t*-test. **p-value ≤ 0.01; ****p-value ≤ 0.0001). **C**, **D** Heatmap of the indicated (**C**) phosphatidylethanolamine (PE) or (**D**) phosphatidylcholine (PC) levels in parental A375 or A375VR cells. Peak area values were normalised to the average of the respective parental values. Only lipids with a median fold change between parental and A375VR cells of p ≤ 0.05 (*n* = 4, two-tailed unpaired *t*-test) are shown. **E** Heatmap showing MAGIC expression z-scores of ferroptosis regulator genes, with cells ordered using hierarchical clustering applied both within and between Seurat clusters, and colour-coded by parental or VR sample identity. **F** Basal lipid peroxidation in A375 and A375VR cells assessed through Alexa Fluor™ 488 fluorescence imaging (*n* = 6, Mean ± SEM, two-tailed unpaired *t*-test. **p-value ≤ 0.01). **G** Total levels (integrated peak areas) of glutathione (GSH) in A375 versus A375VR cells (*n* = 4, Mean ± SEM, two-tailed unpaired *t*-test. ****p-value ≤ 0.0001). **H** Uniform Manifold Approximation and Projection (UMAP) visualisation of parental A375 and A375VR cells coloured by the expression of ferroptosis regulators shown in (**E**) or the expression of the Tsoi undifferentiated (UD) state [5]. **I** Discrete expression of the Tsoi UD state as well as of FAO regulators based on the 90th percentile of signature scores across the whole dataset. **J** Biological functions enriched (based on hypergeometric tests) in the top 200 most overexpressed genes of FAO-high cells as defined (**I**) compared to the remaining cells. The dashed line indicates *p* = 0.05.

Experiments to assess how RANO mediates growth suppression during establishment of BRAFi resistance in melanoma cells revealed a rescue effect by the ferroptosis inhibitors liproxstatin-1 or ferrostatin-1 (Supplementary Fig. 1A, B). This was also seen in BRAFi resistant A375VR cells (Fig. 1A), in which of FAO regulators expression is increased (Supplementary Fig. 1C). Inhibiting caspase-3 driven apoptosis had no effect (Supplementary Fig. 1D), suggesting that RANO suppresses the propagation of BRAFi resistant cells predominantly through the induction of ferroptosis.

To unveil the role of fatty acid metabolism in BRAFi resistance and ferroptosis, we performed metabolomics, lipidomics and transcriptomics analyses of A375VR and A375 parental cells. This revealed a significant increase in PUFA levels in A375VR cells (Fig. 1B), and an increase in PUFA incorporation into phosphatidylethanolamines (PE) and phosphatidylcholines (PC), particularly arachidonic (20:4) and adrenic (22:4) acid, which was accompanied by decreased incorporation of monosaturated fatty acids (MUFAs), such as oleic acid (18:1) (Fig. 1C, D).

scRNAseq analysis identified crucial ferroptosis initiators profoundly enriched in five A375VR-specific Seurat clusters (Fig. 1E, Supplementary Fig. 1E), but individual ferroptosis surveillance factors were also enriched across distinct ACSL4/LPCAT3-high clusters (Fig. 1E). While this implies that the cells engage additional surveillance mechanisms in order to evade ferroptosis, the increased level of lipid peroxidation (Fig. 1F) suggests that A375VR cells are poised to execute ferroptosis.

A moderate enrichment of ferroptosis promoters was also detectable in two A375 cell clusters, but the A375 population was generally enriched in GPX4 and the system Xc^-^-components SLC7A11 and SLC3A2 (Fig. 1E), the latter being in line with higher glutathione (GSH) levels in A375 cells (Fig. 1G).

Mapping the expression of the ferroptosis regulators (inducers, surveillance regulators) onto Seurat clusters (Supplementary Fig. 1E) confirmed enrichment not only in VR cells, but also intermediate expression levels in most parental cells (Fig. 1H). The latter is in line with ferroptosis being elevated in mesenchymal phenotype cancer cells [34] and A375 cells, like all melanoma cells being of neural crest origin. Accordingly, A375 cells are enriched in cells of the Rambow ‘neural stem cell like’ (NCSC) and the Tsoi NC signature [2, 5] (Supplementary Fig. 1E). Nevertheless, the highest level of ferroptosis in melanoma cells has been linked to the Tsoi UD state [5], which is also detectable in A375VR cells and coincides with the highest enrichment of ferroptosis markers (Fig. 1H). Intriguingly, UD-state cells are also highly enriched in FAO regulators (FAO ‘high’) (Fig. 1I), and analysis of FAO-‘high’ cells identifies biological functions characteristic for the UD-state (Fig. 1J). Likewise, in a panel of BRAFi and BRAFi/MEKi resistant melanoma cell lines, a positive correlation of ferroptosis and FAO markers with UD-state markers such as SOX9^high^ and AXL^high^ [5] is seen (Supplementary Fig. 1F).

### Ferroptosis and FAO markers correlate in progressed tumours from patients on BRAFi

**Fig. 2:**
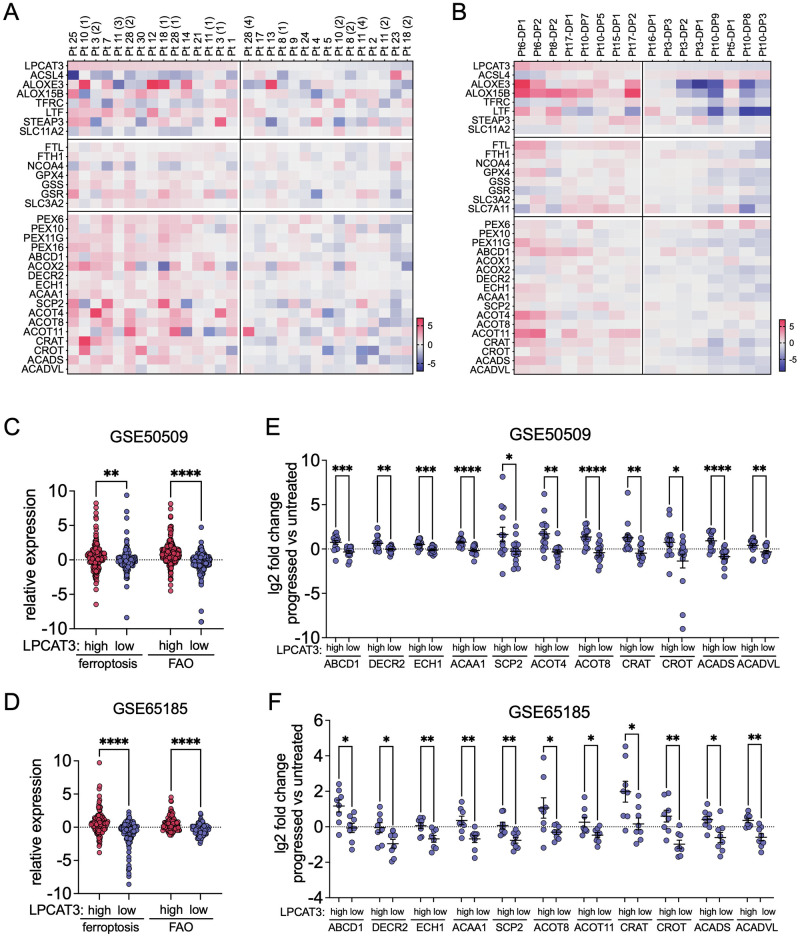
Ferroptosis and FAO marker expression in progressed tumours from patients on BRAFi. **A**, **B** Heatmap of analysis of publicly available gene expression datasets (**A**) GSE50509 [31] and (**B**) GSE65185 [32]. Log2 fold change (FC) in expression of the indicated genes before and after resistance development stratified by the upper and lower median (**A**) or quartile (**B**) of LPCAT3 expression. **C**, **D** Comparative analysis of the log2 FC of all ferroptosis and FAO regulator genes in tumour datasets from (**C**) GSE50509 or (**D**) GSE65185 stratified for high or low LPCAT3 expression (Median, Holm-Sidák test of one-way ANOVA. **p-value ≤ 0.01; ****p-value ≤ 0.0001). **E**, **F** Comparative analysis of log2 FC of the individual indicated genes as in (**C**) and (**D**). (Median, one-way ANOVA with unpaired *t*-test with Welsh correction. *p-value ≤ 0.05; **p-value ≤ 0.01; ***p-value ≤ 0.001; ****p-value ≤ 0.0001).

To validate the relevance of the findings from our in vitro system in a clinical setting, we interrogated tumour expression data from two cohorts of melanoma patients, who had progressed on BRAFi (GSE50509, GSE85185). In particular, we were analysing changes in the expression of markers for pro-ferroptotic activities, ferroptosis surveillance as well as FAO in resistant tumours, when compared to samples from before treatment (Fig. 2A, B). In each cohort adjusting for the expression level of the ferroptosis initiator LPCAT3 identified two groups, in which the expression of these markers was higher or lower in resistant tumours (Fig. 2A, B). Importantly, the tumours that displayed higher expression of ferroptosis markers also exhibited significantly higher expression of FAO markers (Fig. 2C, D), and the changes in expression of LPCAT3 seen in resistant tumours significantly correlated with changes in individual FAO regulator genes (Fig. 2E, F).

### FAO inhibition in BRAFi resistant melanoma cells increases PUFA membrane incorporation and induces ferroptosis

**Fig. 3:**
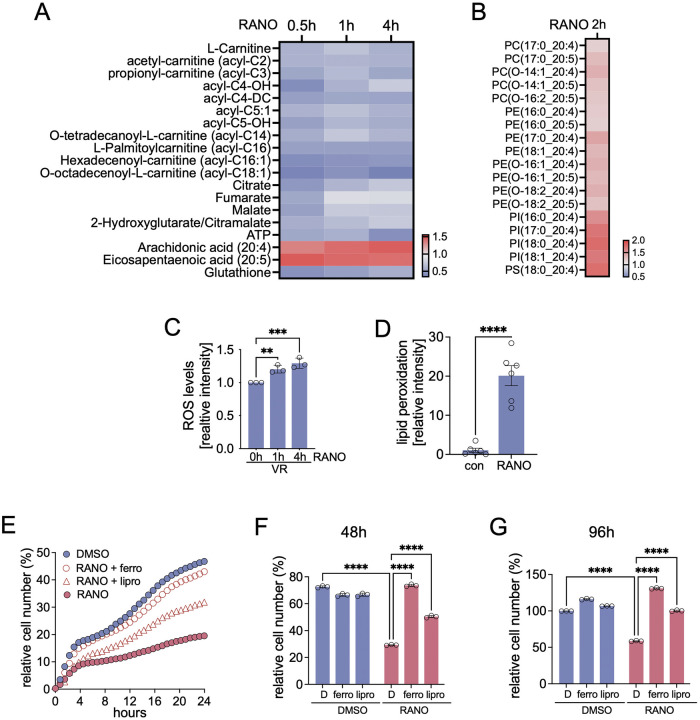
RANO increases PUFA membrane incorporation and induces ferroptosis. **A** Heatmap of the median FC in peak area values (*n* = 8, two-tailed unpaired *t*-test, cut-off *p*-value ≤ 0.05) of the indicated metabolites in A375VR cells treated with RANO for the indicated times relative to DMSO. **B** Heatmap of the median FC in peak area values (*n* = 8, two-tailed unpaired *t*-test, cut-off *p*-value ≤ 0.05) of the indicated phospholipids in A375VR cells treated with RANO for 2 h relative to DMSO. **C** ROS levels in A375VR cells treated with RANO for the indicated times assessed through fluorescence imaging (*n* = 3, Mean ± SD, Dunnett test of one-way ANOVA. **p-value ≤ 0.01; ***p-value ≤ 0.001). **D** Lipid peroxidation in A375VR cells treated with DMSO or RANO assessed through fluorescence imaging (*n* = 6, Mean ± SEM, two-tailed unpaired *t*-test. ****p-value ≤ 0.0001). **E** Real-time cell analysis of A375VR cells treated as indicated. **F,** **G** Relative cell number of A375VR cells treated as indicated for (**F**) 48 h and (**G**) 96 h. (*n* = 3, Mean ± SD, Tukey test of one-way ANOVA. ****p-value ≤ 0.0001).

Our data imply that FAO plays a relevant role in managing ferroptosis in the context of BRAFi resistance. To dissect at the molecular level how RANO impacts on fatty acid metabolism and ferroptosis in BRAFi resistant cells, we analysed the immediate metabolic changes induced by RANO in A375VR cells. Within 30 min of treatment with RANO we observed a significant drop in the levels of acylcarnitines, central TCA cycle metabolites and ATP and these changes were maintained over the 4 h of our analysis, and this was paralleled by increased levels of the PUFAs arachidonic (20:4) and eicosapentaeonic acid (20:5) (Fig. 3A). Lipidomic analysis revealed enhanced incorporation of these respective PUFAs into membrane lipids, particularly PE and PC, as early as 2 h after RANO addition (Fig. 3B). This was accompanied by reduced GSH levels (Fig. 3A), increased ROS levels (Fig. 3C) and a profound increase in lipid peroxidation (Fig. 3D). Overall, RANO induced a significant reduction in cell propagation within 4 h (Fig. 3E), which was rescued by ferrostatin and liproxstatin for up to 96 h (Fig. 3F, G). Together, RANO reduces mitochondrial fatty acid breakdown and GSH levels, which consequently increases PUFA membrane lipid incorporation and lipid peroxidation and ultimately induces ferroptosis.

### Expression of ferroptosis surveillance regulators is suppressed by RANO but maintained in BRAFi-RANO resistant tumours

**Fig. 4:**
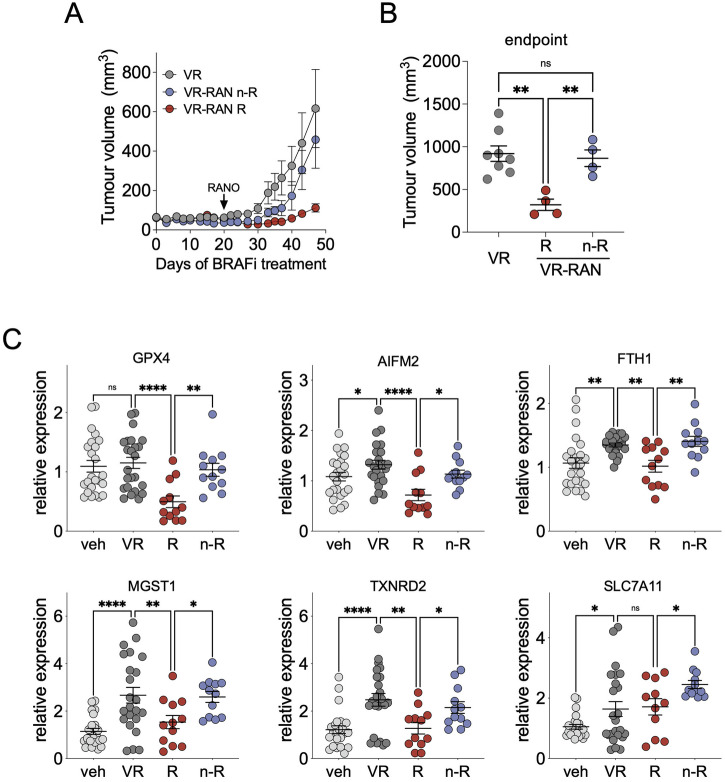
Expression of ferroptosis surveillance markers in BRAFi-RANO resistant tumours. **A** Growth curves of A375 tumours from mice treated with BRAFi (25 mg/kg, daily) and RANO (50 mg/kg, daily) as described [18]. Tumours had been allowed to grow until resistance against BRAFi (VR, *n* = 8) or against BRAFi + RANO (VR-RAN n-R, *n* = 4) was established. Tumours that still responded to BRAFi + RANO were classified as VR-RAN R (*n* = 4). **B** Tumour volume at endpoint of the experiment. (Mean ± SEM, Tukey test of one-way ANOVA. **p-value ≤ 0.01). **C** RT-qPCR analysis of the indicated genes in A375 tumours from mice treated as indicated. Data are triplicates from *n* = 8 tumours for vehicle (veh) or BRAFi (VR), and *n* = 4 tumours for RANO responder (R) or RANO non-responders (n-R). (Mean ± SEM, uncorrected Fisher’s LSD test of one-way ANOVA. *p-value ≤ 0.05; **p-value ≤ 0.01; ****p-value ≤ 0.0001).

We next analysed A375 tumours from mice that had been treated with BRAFi and RANO [18]. In the VR group, animals had been given BRAFi until resistance was established (Fig. 4A and Supplementary Fig. 2A). In the VR-RAN group BRAFi-treated animals received RANO at the first sign of tumour-regrowth. During the following weeks significant growth suppression occurred in RANO responders, while in RANO non-responders, tumour growth eventually recovered (Fig. 4A). At the endpoint of the experiment, tumours were assigned RANO responders (R) or non-responders (n-R) (Fig. 4B).

In tumours from RANO responders (R), we confirmed significantly suppressed expression of FAO regulators compared to VR tumours (Supplementary Fig. 2B). Importantly, also the expression of a number of ferroptosis surveillance regulators was reduced in RANO responding tumours (Fig. 4C). However, in non-responding tumours (n-R), the expression of regulators of FAO as well as of ferroptosis surveillance was restored (Fig. 4C and Supplementary Fig. 2B).

### BRAFi-RANO resistant cells overcome ferroptosis independent of FAO and system Xc^-^

**Fig. 5:**
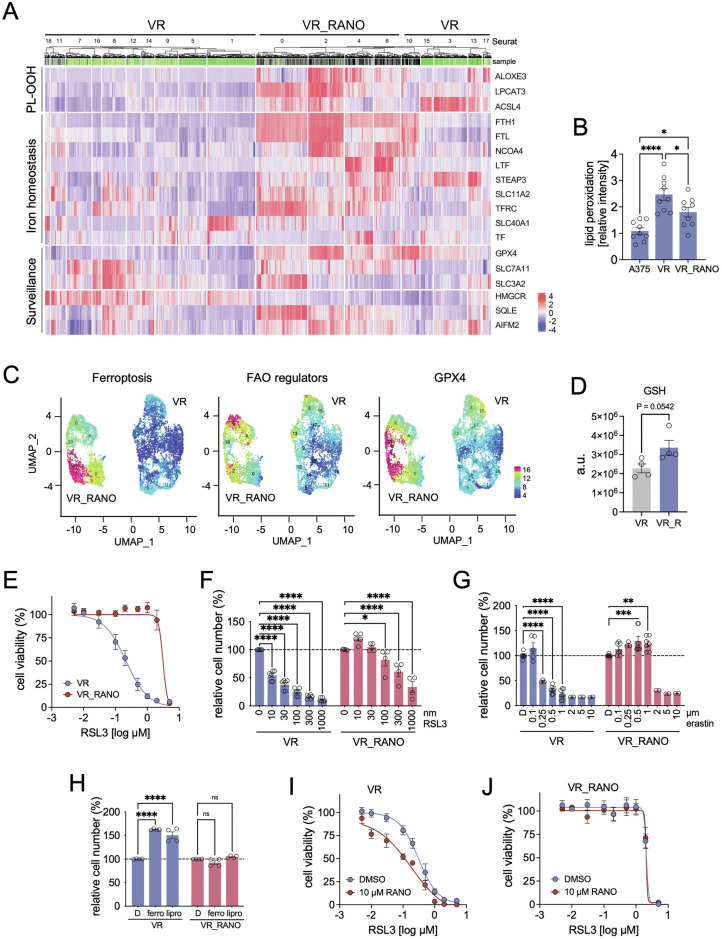
VR_RANO cells are resistant to FAO and system Xc^-^ inhibition. **A** Heatmap showing MAGIC expression z-scores of ferroptosis regulator genes, with cells ordered using hierarchical clustering applied both within and between Seurat clusters, and colour-coded by VR or VR_RANO sample identity. **B** Basal lipid peroxidation in A375, VR and VR_RANO cells assessed through Alexa Fluor™ 488 fluorescence imaging (*n* = 8, Mean ± SEM, Tukey test of one-way ANOVA. *p-value ≤ 0.05; ****p-value ≤ 0.0001). **C** UMAP plot for VR and VR_RANO cells coloured by the expression of ferroptosis regulators, FAO regulators and GPX4. **D** Total levels (integrated peak areas) of glutathione (GSH) in VR versus VR-RANO cells (*n* = 4, Mean ± SEM, two-tailed unpaired *t*-test. *p*-value = 0,0542). **E** Dose response curve for RSL3 in VR and VR_RANO cells. **F**, **G** CFA quantification of VR and VR_RANO cells treated with the indicated concentrations of (**F**) RSL3 and (**G**) erastin. (*n* = 6, Mean ± SEM, Holm-Sidák test of one-way ANOVA. *p-value ≤ 0.05; **p-value ≤ 0.01; ***p-value ≤ 0.001; ****p-value ≤ 0.0001). **H** CFA quantification of VR and VR-RANO cells treated with DMSO (D) or ferrostatin-1 (ferro) or liproxstatin-1 (lipro). (*n* = 4, Mean ± SEM, Holm-Sidák test of one-way ANOVA. ****p-value ≤ 0.0001). **I,** **J** Dose response curve for RSL3 in (**I**) VR and (**J**) VR_RANO cells in the presence or absence of 10 µM RANO.

Our data suggested that BRAFi-RANO non-responder tumours had reinstated activities furthering ferroptosis. Indeed, single cell transcriptomics analysis of resistant VR_RANO cells compared to VR cells confirmed the up-regulation of ACSL4, LPCAT3, and ALOXE3 as well as regulators raising intracellular Fe^2+^ levels (Fig. 5A). This was contrasted by the up-regulation of ferroptosis surveillance regulators, most prominently GPX4 and system Xc^-^ components SLC7A11 and SLC3A2 (Fig. 5A).

Intriguingly, despite higher levels of ACSL4, LPCAT3, and ALOXE3, lipid peroxidation activity was lower in VR_RANO cells compared to VR cells although still higher than A375 (Fig. 5B). This could be a consequence of up-regulated FAO. However, when mapping the enrichment of FAO and ferroptosis markers at single cell level onto the VR_RANO compartment, the respective Seurat clusters did not overlap (Fig. 5C), as previously seen in A375VR cells.

On the other hand, GPX4 was highly enriched and overlapped with ‘ferroptosis marker’ enriched cells (Fig. 5C) and GSH was up-regulated in VR_RANO cells (Fig. 5D). However, while VR cells responded, VR_RANO cells were not only resistant to the GPX4 inhibitor RSL3, but also to erastin induced ferroptosis (Fig. 5E, G), suggesting that these cells do not solely rely on the system Xc^-^-GSH-GPX4 axis to counteract ferroptosis. Furthermore, while as previously observed ferrostatin and liproxstatin, which block alkoxyl radicals, increase cell numbers in VR cells, they had no effect on VR_RANO cells (Fig. 5H). Finally, in VR cells RSL3 acted synergistically with RANO, but VR_RANO cells were resistant to both (Fig. 5I, J). While this confirms the relevance of FAO for BRAFi resistant cells in the context of ferroptosis, it indicates that in BRAFi-RANO resistant cells FAO is not critical in this context.

### BRAFi-RANO resistant cells display MBOAT1/2 mediated phospholipid remodelling

**Fig. 6:**
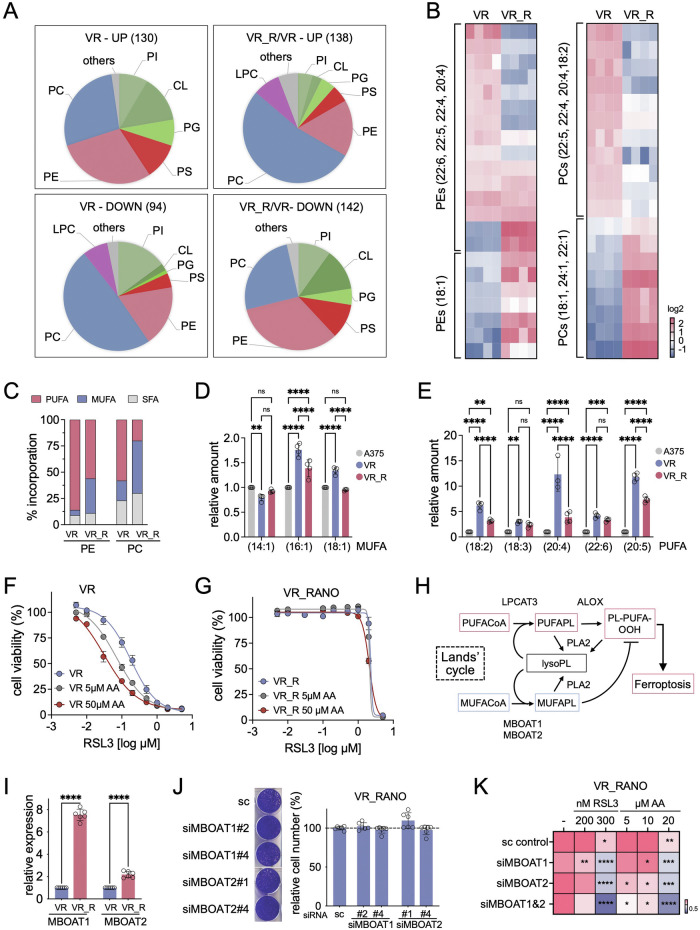
VR_RANO cells display MBOAT1/2 mediated phospholipid remodelling. **A** Quantification of differences in phospholipid levels between VR and VR_RANO cells. The median fold change (*p* < 0.05, two-tailed unpaired *t*-test) from *n* = *4* peak area values between parental and VR and between VR and VR_R samples were assessed for up or down-regulation. PE phosphatidylethanolamine, PC phosphatidylcholine, LPC lysophosphatidylcholine, CL cardiolipin, PG phosphatidylglycerol, PS phosphatidylserine, PI phosphatidylinositol. **B** Heatmap of the indicated phosphatidylethanolamine (PE) or phosphatidylcholine (PC) levels in VR or VR_RANO cells. Peak area values were normalised to the average of the respective VR values. Only lipids with a median fold change between VR and VR_RANO cells with p ≤ 0.05 (*n* = 4, two-tailed unpaired *t*-test) are shown. **C** Relative incorporation of MUFAs, PUFAs and saturated fatty acids (SFAs) into PE and PC in VR or VR_RANO cells considering the median fold change of peak area values (p ≤ 0.05, *n* = 4, two-tailed unpaired *t*-test). **D**, **E** Relative levels of the indicated (**D**) MUFAs and (**E**) PUFAs in A375, VR and VR_RANO cells; A375 cells were set 1. (*n* = 4, Mea*n* ± SEM, Mean ± SD, Holm-Sidák test of 2-way ANOVA. **p-value ≤ 0.01; ***p-value ≤ 0.001; ****p-value ≤ 0.0001). **F**, **G** Dose response curve for RSL3 in (**F**) VR and (**G**) VR_RANO cells in the presence or absence of 5 µM or 50 µM arachidonic acid (AA). **H** Schematic of the action of MBOAT1/2 in the Lands’ Cycle. **I** RT-qPCR for MBOAT1 and MBOAT2 in VR and VR_RANO cells. (*n* = 6, Mea*n* ± SD, uncorrected Fisher’s LSD test of one-way ANOVA. ****p-value ≤ 0.0001). **J** CFA quantification of VR-RANO cells transfected with a control (sc) siRNA or siRNAs targeting MBOAT1 or MBOAT2. (*n* = 6, Mea*n* ± SEM, Holm-Sidák test of one-way ANOVA). **K** Heatmap of CFA quantification of VR-RANO cells transfected as described and treated with the indicated concentrations of RSL3 or arachidonic acid (AA). (*n* = 3, Mea*n* ± SEM, Mean ± SEM, Holm-Sidák test of 2-way ANOVA. *p-value ≤ 0.05; **p-value ≤ 0.01; ***p-value ≤ 0.001; ****p-value ≤ 0.0001).

To better understand why VR_RANO cells display reduced lipid peroxidation independent of FAO and system Xc^-^, we performed a lipidomics analysis in order to assess the phospholipid content in VR and VR_RANO cells. This revealed quantitative changes in VR_RANO compared to VR cells, particularly in PE and PC (Fig. 6A). A more detailed analysis revealed reduced levels of phospholipids containing arachidonic acid (20:4, AA) in VR-RANO cells, whereas incorporation of the MUFA oleic acid (18:1) was increased (Fig. 6B). Overall, in VR_RANO cells PUFA incorporation into PE and PC was reduced and MUFA incorporation increased (Fig. 6C). Nevertheless, there was no increase in cellular MUFA levels, but levels of PUFAs in VR_RANO cells, particularly of AA were reduced (Fig. 6D, E).

Reduced PUFA synthesis and as such reduced cellular levels can lead to resistance to RSL3, but this can be overcome by AA supplementation [35]. Indeed, in A375VR cells addition of AA increased the RSL3 inhibitory effect, but this had no significant effect on RSL3 action in VR_RANO cells (Fig. 6F, G). Thus, in VR_RANO cells incorporation of MUFAs, particularly oleic acid might be favoured over AA incorporation due to changes in acyl transferase activities.

Lysophospholipid acyltransferases that preferentially incorporate oleic acid into phospholipids in the context of the Lands’ cycle are MBOAT1 and MBOAT2 [36] (Fig. 6H), both of which are up-regulated in VR_RANO cells (Fig. 6I). Depletion of MBOAT1 or MBOAT2 alone had no significant effect on cell propagation (Fig. 6J), but it significantly sensitised VR_RANO cells to RSL3 and AA, and this was seen more pronounced when both MBOATs were depleted (Fig. 6K and Supplementary Fig. 3A–D),

### AR regulates MBOAT1/2 and contributes to ferroptosis surveillance in BRAFi resistant melanoma cells

**Fig. 7:**
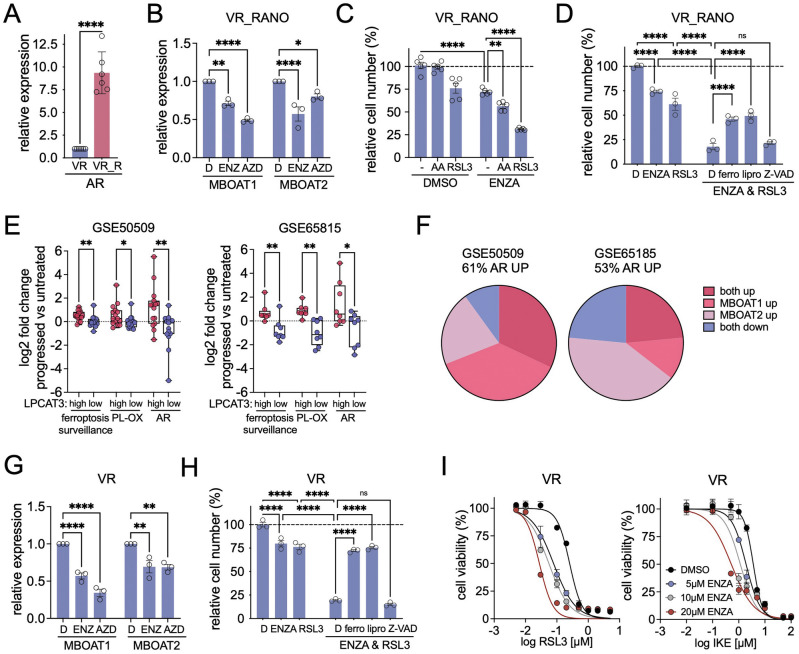
AR regulates MBOAT1/2 expression and contributes to ferroptosis surveillance. **A** RT-qPCR for AR in VR and VR_RANO cells. (*n* = 6, Mean ± SD, two-tailed unpaired *t*-test). ****p-value ≤ 0.0001. **B** RT-qPCR for MBOAT1 and MBOAT2 in VR_RANO cells treated with 20 µM enzalutamide (ENZ) or 20 µM AZD3514 (AZD). (*n* = 3, Mean ± SEM, Sidák test of one-way ANOVA. *p-value ≤ 0.05; **p-value ≤ 0.01; ****p-value ≤ 0.0001). **C** CFA quantification of VR-RANO cells treated with DMSO or enzalutamide (ENZA) in the absence or presence of RSL3 or AA (*n* = 5, Mean ± SEM, Holm-Sidák test of one-way ANOVA. **p-value ≤ 0.01; ****p-value ≤ 0.0001). **D** CFA quantification of VR-RANO cells treated with DMSO (D), ENZA or RSL3 alone or in combination in the absence or presence of ferrostatin-1 (ferro), liproxstatin-1 (lipro) or Z-VAD-FMK. (*n* = 3, Mean ± SEM, Holm-Sidák test of one-way ANOVA. ****p-value ≤ 0.0001). **E** Comparative analysis of the log2 FC of AR and ferroptosis surveillance and lipid peroxidation (PL-OX) regulator genes in tumour datasets stratified for high or low LPCAT3 expression. GSE50509 (upper lower Median, *n* = 30), GSE65185 (upper lower quartile, *n* = 16). (One-way ANOVA with unpaired *t*-test with Welsh correction. *p-value ≤ 0.05; **p-value ≤ 0.01). **F** Expression changes of MBOAT1 and MBOAT2 in AR up-regulated BRAFi resistant tumours represented in GSE50509 and GSE65185. **G** RT-qPCR for MBOAT1 and MBOAT2 in VR cells treated 20 µM enzalutamide (ENZ) or 20 µM AZD3514 (AZD). (*n* = 3, Mean ± SD, Sidák test of one-way ANOVA. **p-value ≤ 0.01; ****p-value ≤ 0.0001). **H** CFA quantification of VR cells treated with DMSO (D), ENZA or RSL3 alone or in combination in the absence or presence of ferrostatin-1 (ferro), liproxstatin-1 (lipro) or Z-VAD-FMK. (*n* = 3, Mean ± SEM, Holm-Sidák test of one-way ANOVA. ****p-value ≤ 0.0001). **I** Dose response curve for RSL3 or imidazole ketone erastin (IKE) in VR cells in the presence or absence of the indicated concentrations of ENZA.

*MBOAT2* expression is regulated by the androgen receptor (AR) through direct binding to an intronic androgen response element (ARE) [37] and an ARE with proximity to the transcription start site is also found in *MBOAT1* [38, 39]. VR_RANO cells display profoundly increased expression of AR (Fig. 7A), and we confirmed the binding of AR to the corresponding intervals in *MBOAT1* as well as *MBOAT2*, which was further increased after addition of Dihydrotestosterone (DHT) (Supplementary Fig. 4A–D). Accordingly, AR activation by DHT up-regulated MBOAT1 and MBOAT2 transcript levels (Supplementary Fig. 4E), and AR inhibition by enzalutamide (ENZA) or AZD3514 (AZD) reduced MBOAT2 and MBOAT1 expression (Fig. 7B), further substantiating the link between AR activation and these lysophospholipid acyltransferases in VR_RANO cells.

ENZA increased the responsiveness of VR_RANO cells to AA and RSL3 (Fig. 7C), which could be rescued by the addition of ferrostatin and liproxstatin, but not by a pan caspase inhibitor (Fig. 7D), revealing an involvement of AR in ferroptosis surveillance. Because AR is up-regulated in BRAFi resistant melanoma cells as well as in BRAFi/MEKi treated patients, where this correlates with lower pathological response rates [40, 41], we assessed the relevance of our finding in melanomas that had progressed in BRAFi treated patients.

We found that AR up-regulation significantly correlated with LPCAT3 expression, and markers of ferroptosis surveillance and phospholipid-peroxidation (Fig. 7E). AR expression was upregulated in 53-61% of progressed tumours, and in 75-90% of these AR-positive tumours MBOAT1 and MBOTA2 expression was increased (Fig. 7F). There were no sex-specific differences in progressed tumours with upregulated AR with regard to basal expression or fold change in progressed versus untreated tumours (Supplementary Fig. 5A–F).

In BRAFi resistant A375VR cells AR inhibition reduces MBOAT1 and MBOAT2 expression (Fig. 7G) and ENZA synergises with RSL3 to induce cell death, which can be rescued by ferrostatin and liproxstatin (Fig. 7H). The sensitisation to ferroptosis induction by ENZA was dose-dependent and was also seen with the system Xc^-^ inhibitor imidazole ketone erastin (IKE) (Fig. 7I).

### AR and FAO inhibition synergise in killing BRAFi resistant melanoma cells

**Fig. 8:**
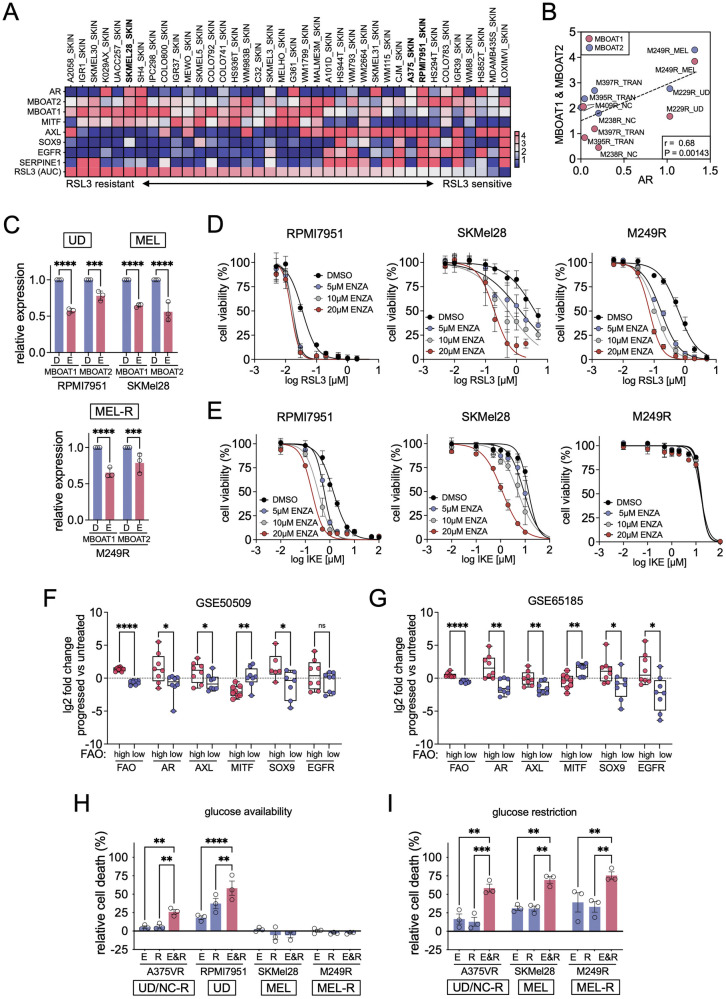
AR and FAO inhibition sensitise to ferroptosis in BRAFi resistant melanoma cells. **A** Heatmap of the expression (RPKM) of the indicated genes in the BROAD cell line panel sorted for RSL3 sensitivity (area under curve; AUC). AUC values were obtained from the Cancer Therapeutics Response Portal (CTRP); http://portals.broadinstitute.org/ctrp/. **B** Correlation of AR with MBOAT1/2 expression in the Tsoi panel [5] of BRAFi resistant melanoma cell lines. The transcriptional state of each cell line is indicated. **C** RT-qPCR for MBOAT1 and MBOAT2 in the indicated cell lines treated with DMSO (D) or enzalutamide (E). (*n* = 3, Mean ± SD, Sidák test of one-way ANOVA. ***p-value ≤ 0.001; ****p-value ≤ 0.0001). **D**, **E** Dose response curve for (**D**) RSL3 or (**E**) imidazole ketone erastin (IKE) in the indicated cell lines in the presence or absence of the indicated concentrations of ENZA. **F**, **G** Comparative analysis of the log2 FC of FAO regulators, AR and the indicated UD state markers in tumour datasets (**F**) GSE50509 and (**G**) GSE65185 stratified for high or low FAO regulator expression. (upper and lower quartile, one-way ANOVA with unpaired t-test with Welsh correction. *p-value ≤ 0.05; **p-value ≤ 0.01; ****p-value ≤ 0.0001). **H**, **I** Relative cell death quantification in the indicated cell lines (**H**) in glucose availability conditions (25 mM glucose) or (**I**) under glucose restriction conditions (5 mM glucose). Cells had been treated with ENZA (E), RANO (R) or the combination of both (E&R). (*n* = 3, Mean ± SEM, Holm-Sidák test of one-way ANOVA. *p-value ≤ 0.05; **p-value ≤ 0.01; ****p-value ≤ 0.0001).

We find that AR and MBOAT1/2 contribute to ferroptosis surveillance in A375VR cells, which display a dedifferentiated UD/NC state (see Fig. 1H and Supplementary Fig. 1E). Of note, cells of this phenotype state are generally more sensitive to ferroptosis induction independently of BRAFi resistance [5, 42]. Accordingly, in the CCLE panel of naïve melanoma cell lines RSL3 sensitivity correlates with the expression of markers of the UD/NC state such as AXL, SOX9, EGFR, and SERPINE1 (Fig. 8A). In contrast, higher MBOAT1/2 expression correlated with resistance to RSL3, and for MBOAT1 this reached significance (Fig. 8A and Supplementary Fig. 6A). Nevertheless, while AR expression was more prominent in AXL^high^ UD/NC cells, it was also frequently seen in MITF^high^ melanocytic (MEL) cells (Supplementary Fig. 6B) and there was no apparent correlation with MBOAT1/2 expression. In the Tsoi panel of naïve melanoma cells [5], cells of the UD state displayed a slightly higher expression of AR compared to the MEL state, but this also did not correlate with MBOAT1/2 expression (Supplementary Fig. 6C). Overall, this suggests that in naïve melanoma cells AR expression, despite more abundant in cells of the UD/NC state is also detectable in cells of the MEL state, and additional factors apart from AR regulate the expression of MBOAT1/2.

AR up-regulation in BRAFi resistant melanoma cells has been related to the UD/NC state [40], and in the Tsoi panel of resistant cell lines we confirm that AR-up-regulation correlates with increased AXL, SERPINE1 and EGFR expression. However, this was also seen in resistant transitory (TRAN) state M397R cells (Supplementary Fig. 6D). Moreover, the actual expression levels of AR in BRAFi resistant cells, which correlated with MBOAT1 and MBOAT2 expression, were not only elevated in cells of the UD/NC state, but also in cells of the TRAN state and in M249R cells of the MEL state (Fig. 8B).

Together, although AR expression appears to be predominantly linked to the UD/NC state, it might also play a role in ferroptosis surveillance in cells of the MEL state. In line with such a general role in ferroptosis surveillance, AR regulates MBOAT1/2 expression in RPMI7951 UD cells, in SKMEL28 MEL cells, as well as resistant melanocytic M249R (MEL-R) cells (Fig. 8C), and ENZA sensitises RPMI7951, SKMEL28, and M249R cells to ferroptosis induction by RSL3 as well as IKE (Fig. 8D, E).

In BRAFi relapsed tumours from melanoma patients, stratification for AR upregulation correlated with AXL or EGFR but did not significantly separate for further markers of the UD state (Supplementary Fig. 6E). However, considering FAO regulators resulted in significant separation of tumours with up- or down-regulation of AR as well as UD markers (Fig. 8F, G). In line with this, ENZA and RANO together increased cell death in RPMI7951 and A375VR cells, but SKMEL28 and M249R cells were unaffected (Fig. 8H). Moreover, lowering glucose availability, which pushes melanoma cells towards the use of FAO [18] and induces a UD/NC phenotype [43], sensitised these MEL cell lines to combined AR and FAO inhibition (Fig. 8I).

## Discussion

Inducing ferroptosis can significantly improve the efficacy of killing tumour cells, particularly of drug-resistant cells that are refractory to drug-induced apoptosis [44]. High MITF expressing melanoma cells of the melanocytic MEL state are poorly responsive to targeted therapy [10, 12], and they are relatively resistant to ferroptosis induction [5], because they possess enhanced intrinsic antioxidant defences controlled by the melanocyte master regulator MITF [45–49]. Importantly however, cells of the AXL^high^ UD/NC state, which are highly resistant to both, targeted and immunotherapy [6, 14, 32, 50] are sensitive to ferroptosis induction through GPX4 or xCT/SLC7A11 inhibition [5].

Drugs inhibiting the system Xc^-^-GSH-GPX4 axis are currently trialled in patients, but resistance to these drugs mediated by other surveillance mechanisms poses a problem [44]. We identify FAO as such a ferroptosis surveillance mechanism in BRAFi resistant cells of the UD/NC state in melanoma, and accordingly inhibiting FAO with RANO enhances ferroptotic activities and sensitises to GPX4 inhibition. Nevertheless, melanoma cells could circumvent ferroptosis induction triggered by FAO inhibition through AR and MBOAT1/2 mediated phospholipid remodelling. A similar mechanism has been observed in prostate cancer cells, where AR controls ferroptosis not only via MBOAT2 [37], but also through other mechanisms [51] involving SLC7A11 [52, 53], PEX10 [54] or ACSM1/3 [55]. Nevertheless, in melanoma AR signalling had so far not been linked to ferroptosis.

Instead, AR has been shown to prevent cellular senescence and genomic DNA damage in melanoma cells, and to drive invasiveness and contribute to melanoma metastasis and progression [56–58]. While all these activities are in line with the fact that male sex is associated with worse outcomes in patients with melanoma [59, 60], it appears that this cannot be simply linked to sexual dimorphism in AR expression or nuclear localisation [57, 58]. Nonetheless, higher circulating testosterone levels have been associated with increased melanoma risk in male patients [61], hinting to relevance at the level of AR function and signalling. Considering this, a clear sexual dimorphism has been reported in the context of BRAFi/MEKi targeted therapy, where higher rates of major pathological response could be linked to a lower AR signature score accompanied by lower levels of nuclear localisation in tumours from female patients [41]. We found no obvious correlation of patients’ sex origin with AR expression in progressed tumours, but we did not have information on AR nuclear localisation and the datasets we analysed were from patients on BRAFi monotherapy, in which sexual dimorphism appears to be less prominent [41].

Inhibiting AR with enzalutamide improves BRAFi/MEKi therapy in mice [41] and reduces the growth of BRAFi resistant melanoma xenografts [40]. We find that apart from this basal anti-tumour activity, enzalutamide sensitises not only BRAFi resistant cells, but also naïve melanoma cells to ferroptosis; and this ability of enzalutamide is independent of melanoma phenotype/cell state. Nevertheless, in the context of BRAFi therapy AR up-regulation correlates with a shift towards the UD/NC state [40], which is poised to ferroptosis execution [5]. This suggests these cells are more dependent on an AR mediated ferroptosis surveillance, making them a perfect target for enzalutamide. Moreover, we find that FAO is enriched in cells of the UD/NC state, and because AR cooperates with FAO in ferroptosis surveillance, an enzalutamide/RANO combination could further enhance the killing effect.

Effectively targeting UD/NC state cells is not only relevant for targeted therapy but also for immunotherapy, where they contribute to the establishment of an immunosuppressive environment [62, 63]. Both, FAO and ferroptosis play complex roles in the immune response, whereby FAO activity is linked to T cell activation versus persistence [64] and cancer cells poised to ferroptosis display increased sensitivity to cytotoxic T cells [65, 66]. Enzalutamide can increase the sensitivity of prostate cancer cells to T-cell-mediated killing in vitro [67], but its effects on the immune-microenvironment are complex [68, 69]. However, in vivo it can sensitise to anti-PD-1 therapy in a prostate cancer model [70] and it can trigger an enhancement of clustered CD8 + T-cell infiltration in a melanoma model [40]. Importantly, RANO increases intra-tumoral cytotoxic T cell abundance, reduces their exhaustion and improves the anti-PD-L1 therapy response [18]. Thus, with AR and FAO counteracting ferroptosis execution in melanoma cells, combining enzalutamide and RANO might represent a therapeutic option to not only delay the onset of BRAFi acquired resistance but also to improved responses to immunotherapy.

## Supplementary information

Supplementary information revised

## Data Availability

scRNA-seq data of human melanoma cell lines are deposited in ArrayExpress under accession number E-MTAB-12412. Metabolomics and lipidomics data are available at the NIH Common Fund’s National Metabolomics Data Repository (NMDR) website https://www.metabolomicsworkbench.org, where the data sets have been assigned Study IDs ST002713 and ST002712 (lipidomics and metabolomics resistant cells), and ST003945 and ST003944 (lipidomics and metabolomics time course).
