# Supplementary material for: Androgen receptor and fatty acid oxidation cooperate in ferroptosis evasion in BRAFi resistant melanoma
Source: Cell Death Dis. 2026 Mar 23;17(1):338. doi: 10.1038/s41419-026-08578-4 (PMC13039702; doi:10.1038/s41419-026-08578-4)
Supplement: Supplementary file 1 — Supplementary information revised [file 41419_2026_8578_MOESM1_ESM.pdf]

Supplementary Information

Androgen receptor and fatty acid oxidation cooperate in ferroptosis evasion in BRAFi resistant melanoma

Marta Redondo-Muñoz[1,2], Adria Caballe-Mestres[3], Julie A. Reisz[4], Ane Valero-Leria[1,2], Ana Olias-Arjona[1,2], Paula Aldaz[1,2], Angelo D´Alessandro[4], Claudia Wellbrock[1,2,5 *] and Imanol Arozarena[1,2,*]

1. Cancer Signaling Unit, Navarrabiomed, Hospital Universitario de Navarra (HUN), Universidad Pública de Navarra (UPNA), Pamplona, Spain.

2. Health Research Institute of Navarre (IdiSNA), Pamplona, Spain

3. Institute for Research in Biomedicine (IRB Barcelona), The Barcelona Institute of Science and Technology (BIST), Barcelona, Spain

4. Department of Biochemistry and Molecular Genetics, University of Colorado Anschutz Medical Campus, Aurora, CO, USA

5. Department of Health Sciences, Universidad Pública de Navarra (UPNA), Pamplona, Spain

* co- corresponding authors

Correspondence: imanol.arozarena.martinicorena@navarra.es

Table S1 Primers used for real-time PCR

Gene	Forward sequence (5'-3')	Reverse sequence (5'-3')
CPT1A	GACGTGGGAAAAATAAGCAGTC	ACATCGGCCGTGTAGTAGAGAT
ACOX1	GGTTTAAAAATTTTGTGCACCGAGG	CGAAGGTGAGTTCCATGACCC
ACADS	CACGCCTTTCACCAGTGGTGAC	GGCATTGGTGATCCAGGCTTTG
EHHADH	ATAGGATTGCCCACGCAGAG	TGCTAAAATACGTCTTCCTGAGGT
GPX4	GGCGGAAGAAGCCCTGTCC	GCACACGAAGCCCCGGT
AIFM2	GACTCCTTCCACCACAATGTGG	CAGCACCATCTGGTTCTTCAGG
FTH1	TGAAGCTGCAGAACCAACGAGG	GCACACTCCATTGCATTCAGCC
MGST1	AGAACGTGTACGCAGAGCC	GCAATGGTGTGGTAGATCCGT
TXNRD2	GCTGCGGGGATTAGGAGGG	ATCATAGTCCCGCTGACCTGC
SLC7A11	ACAGGGATTGGCTTCGTCAT	TGTTCTGGTTATTTTCTCCGACATT
MBOAT1	GGTTTCCACAGCTTGCCAGAAC	ACCAGTCATCCACAAGGCAGGT
MBOAT2	GGGTATGACGAAAATGGAGCAGC	CTTTTGAGCCAAAGAGCTGTCTG
AR	ATGGTGAGCAGAGTGCCCTATC	ATGGTCCCTGGCAGTCTCCAAA

Supplementary Methods

If not otherwise indicated, in vitro experiments were performed in triplicates (*n* = 3 technical replicates,) and were repeated at least three times in the laboratory.

Cell lines and reagents

A375 (female), RPMI7951 (female) and SKMEL28 (male) melanoma cells were from ATCC; M249R cells (female) were a gift from Dr Antoni Ribas (26). All cell lines had been authenticated in 2021 by STR profiling using the AmpFlSTR™. RPMI7951 cells were cultured in RPMI1640 medium; A375, SKMEL28 and M249R cells were cultured in Dulbecco's Eagle's Medium, all supplemented with 10% fetal bovine serum plus 1% penicillin/streptomycin. Vemurafenib (cat# HY-12057), Ranolazine (cat#HY-17401), Ferrostatin1 (cat#HY-100579), Liproxstatsin-1 (cat#HY-12726), RSL3 (cat#HY-100218A), Imidazole ketone erastin (IKE) (cat#HY-114481), Arachidonic acid (AA) (cat#HY-109590), Enzalutamide (cat#HY-70002), Z-VAD-FMK (cat#HY-16658B), Z-DEV-FMK (cat#HY-12466), Erastin (cat#HY-15763) and AZD3514 (cat#HY-16079) were from MedChemExpress. Dihydrotestosterone (DHT) (cat#A8380) was from Merck.

Acquired resistance establishment protocol

Melanoma cells seeded in 6-well plates were treated for 7 days with 5 µM vemurafenib before switching to 0,5 µM. Fresh medium and vemurafenib was added once weekly for further 3-4 weeks until arising colonies grew to confluence. 100µM ranolazine was added once per week, and ferrostatin-1 or liproxstatin-1 were added as indicated.

Single cell RNA sequencing analysis

The generation of single cell RNAseq data from parental A375 and resistant A375VR and A375VR_RANO cells has been described previously (18). The first 10 principal components of the integrated dataset were used to obtain the UMAP and the clustering of cells with FindClusters Seurat function (resolution = 1.2). SCTransform integrated counts were imputed and smoothed with magic (v.2.0.3) (27). For every cell, gene signatures scores were calculated by taking the average magic expression of their constituent genes. FindAllMarkers was used to identify the most differentially expressed genes with parameters min.pct = 0.25, logfc.threshold = 0.25, and considering as identity classes either Seurat clusters or treatments. For several biological functions, high expressed cells were singled out as cells falling above the 90th percentile of the observed gene signature scores distribution across the whole dataset. Gene markers were obtained for the high expressed populations using Seurat function FindMarkers (min.pct = 0.2, logfc.threshold = 0.2, considering only the top 200 most differentially expressed genes). These most differentially expressed sets were tested for biological enrichment with hypergeometric tests using Hallmarks, GO, KEGG as well as other previously published gene signatures. P-values were corrected for multiple comparisons using the Benjamini–Hochberg approach. The downstream analyses detailed above (from data normalization and integration to biological enrichment) were done independently for two subsets of data: (a) parental + VR cells; and (b) VR + VR_RANO cells.

Code availability

Code used for human melanoma cell scRNAseq analysis is available at:

https://doi.org/10.5281/zenodo.8093417

Metabolomics and lipidomics analyses

The generation of lipidomic and metabolomic data of parental A375 cells and resistant A375VR and A375VR-RANO cells has been described previously (18). For the short-term treatment (time course) A375VR cells (*n* =8 for each time point) were treated with 100 µM RANO for the indicated times and then cells were collected, washed three times with cold PBS, centrifuged and the cell pellets were frozen.

Sample Preparation: Frozen cell pellets were extracted at $2x10^6$ cells/mL with cold 100% MeOH or MeOH:MeCN:H2O (5:3:2, v:v:v) or for lipidomics or metabolomics, respectively. Suspensions were vortexed for 30 min at 4°C. Insoluble material was pelleted by centrifugation (18,213 *g*, 10 min, 4°C) and supernatants were isolated for analysis by UHPLC-MS.

Data acquisition and analysis: A Vanquish UHPLC system (Thermo) was coupled to an Orbitrap Exploris 120 mass spectrometer (Thermo) for metabolomics and a Q Exactive mass spectrometer (Thermo) for lipidomics. Metabolites were resolved across a 2.1 x 150 mm, 1.7 um Kinetex SB-C18 column (Phenomenex) using a 5 minute, reverse-phase gradient from a previously described method (28). Run order of samples was randomized and technical replicates were included to assess quality control. Raw files were converted to mzXML using RawConverter. The resultant files were processed with El-Maven (Elucidata) alongside the KEGG database and an in-house standard library for metabolite assignment and peak integration as previously described (29). Lipidomics analysis employed a Vanquish UHPLC system coupled to a Q Exactive mass spectrometer (Thermo). The samples were randomized and resolved across a 2.1 x 30 mm, 1.7 um Kinetex C18 column (Phenomenex) using a 5 minute reverse-phase gradient with data dependent MS/MS fragmentation as previously described (30). Technical replicates from $2x10^6$ cells/mL were included to assess quality control. Lipid assignments and peak integration were performed using LipidSearch v 5.0.

Colony formation and cell viability assays

Cells seeded in 6-well plates were treated with inhibitors or DMSO 24h after plating. Cells were left to form colonies for 7 to 14 days (depending on the cell line) until control cells had reached an appropriate density. Then, cells were fixed and stained and absorbance measured. For cell viability assays, 5000 cells/well were seeded in 96-well plates, drugs added after 24h and cells were analysed after 72h. Glucose restriction was achieved using medium with no glucose, supplemented with D-(+)-Glucose solution (cat#G8769, Merck) to reach 5mM. RPMI7951 cells were excluded from this analysis as they detached under these conditions.

A375 xenograft analysis

Female Foxn1nu/Foxn1nu mice (8 weeks of age) were subject to approval by the Biodonostia HRI animal experimentation ethics committee. Randomization was used to allocate mice into cages and no blinding was performed. ARRIVE guidelines were followed for animal experimentation. The sample size was calculated using previous information regarding this type of biological assay. In total, 2×10^6 A375 human melanoma cells had been injected as described (18). Once tumours reached ~60 mm^3, mice were randomly assigned to different groups; mice that had not developed any tumour had been excluded from the experiment. Drugs had been administered by intraperitoneal injection. Vehicle, vemurafenib (25 mg per kg body weight, alone or in combination with RANO (50 mg per kg body weight) had been administered once daily for up to 50 d and tumour volumes measured every 3 d. Before tumours reached maximal size (1500 mm^3), they were collected and snap frozen for RNA extraction. For analysis we selected RNA samples corresponding to BRAFi resistant tumours from n = 8 animals, to tumours from animals treated with BRAFi and RANO with a volume below 500 mm^3 (n =4) classified as RANO responder (R), and to samples from BRAFi/RANO

treated animals (*n* =4) with tumours with a size above ≥590 mm^3, designated RANO non-responder (n-R).

Lipid peroxidation and ROS measurements

Lipid peroxidation (basal in the respective cell lines or in A375VR cells, treated with 100 µM RANO for 2h) was measured using the Click-iT™ Lipid Peroxidation Imaging Kit - Alexa Fluor™ 488 from ThermoFisher (C10446) following manufacturer's instructions. For ROS measurements, A375 VR cells were treated with 100 µM RANO for 1-4 hours and ROS production was analysed by using DCFDA/H_2DCFDA-Cellular ROS Assay Kit (ab113851, Abcam Cambridge, UK) according to the manufacturer's instructions. Cells treated with TBHP, a treatment that elicits ROS production, were used as positive control.

XCELLigence Real-Time Cell Analysis

A375VR cells were seeded (5000 cells/well) in E-Plate VIEW 16 plates (Agilent, 300601140) using Agilent xCELLigence Real-Time Cell Analysis (RTCA) technology (Agilent, xCELLigence RTCA DP) for real time cell proliferation analysis. Inhibitors and DMSO were added 24h after plating following the manufacturer's indications and cell growing was recorded until control cells confluence.

RNA isolation and RT-qPCR analysis

For RT-qPCR experiments in cell lines, total RNA from cells was extracted with Trizol (Thermo Fisher). In all cases RNA was DNase treated (Invitrogen) and reverse transcription performed using PrimeScript RT Reagent Kit (Takara). RT-qPCR was performed using SYBR Green (Thermo Fisher) and a QuantStudio 12K Flex qPCR system (Applied Biosystems) with triplicate

biological repeats for each sample, and fold change calculated normalised to 18S expression. The primers used are indicated in Table S1.

Chromatin immunoprecipitation (ChIP)

ChIP experiments were carried out using Pierce™ Magnetic ChIP Kit (Cat# 26157, ThermoFisher). Briefly, according to the manufacturer's protocol, cells were fixed with 1% formaldehyde for 10 min, after which the reaction was quenched by the addition of glycine. The fixed cells were washed with PBS and harvested by scraping in PBS with protease inhibitor cocktail for pellet isolation. Cell lysis and nuclei digestion were performed by enzymatic digestion and sonication to obtain digested chromatin. An input sample (10% of digested chromatin) was collected at this point. Digested chromatin was incubated overnight with primary antibody (Androgen receptor antibody, Cat#06-680, Millipore) and rabbit IgG as negative control. The next day, magnetic beads were incubated with each IP reaction for 2 hours at 4°C with mixing. Beads were washed and collected using a magnetic stand (Cat#12321D, DynaMag™-2 Magnet Invitrogen™) according to the manufacturer procedure. Then, beads were incubated in elution buffer and DNA was purified from each IP reaction. ChIP samples and input DNA were analysed by qPCR using SYBR Green Master Mix (Cat#4312704, Applied Biosystems) and primers against MBOAT1 and MBOAT2 promoters (MBOAT1: ChIP-F1:CTCCAGCAGGAGTGAGTGTG, ChIP-R1:CTTCCAAACTCGCAAGCCAC; MBOAT2: ChIP-F1:GTAGGTTTGGACTGGCAGCA, ChIP-R1:CGTAGCACCACGCATTACTC).

Redondo-Muñoz et al, Supplementary Figure 1
(corresponding to Figure 1)

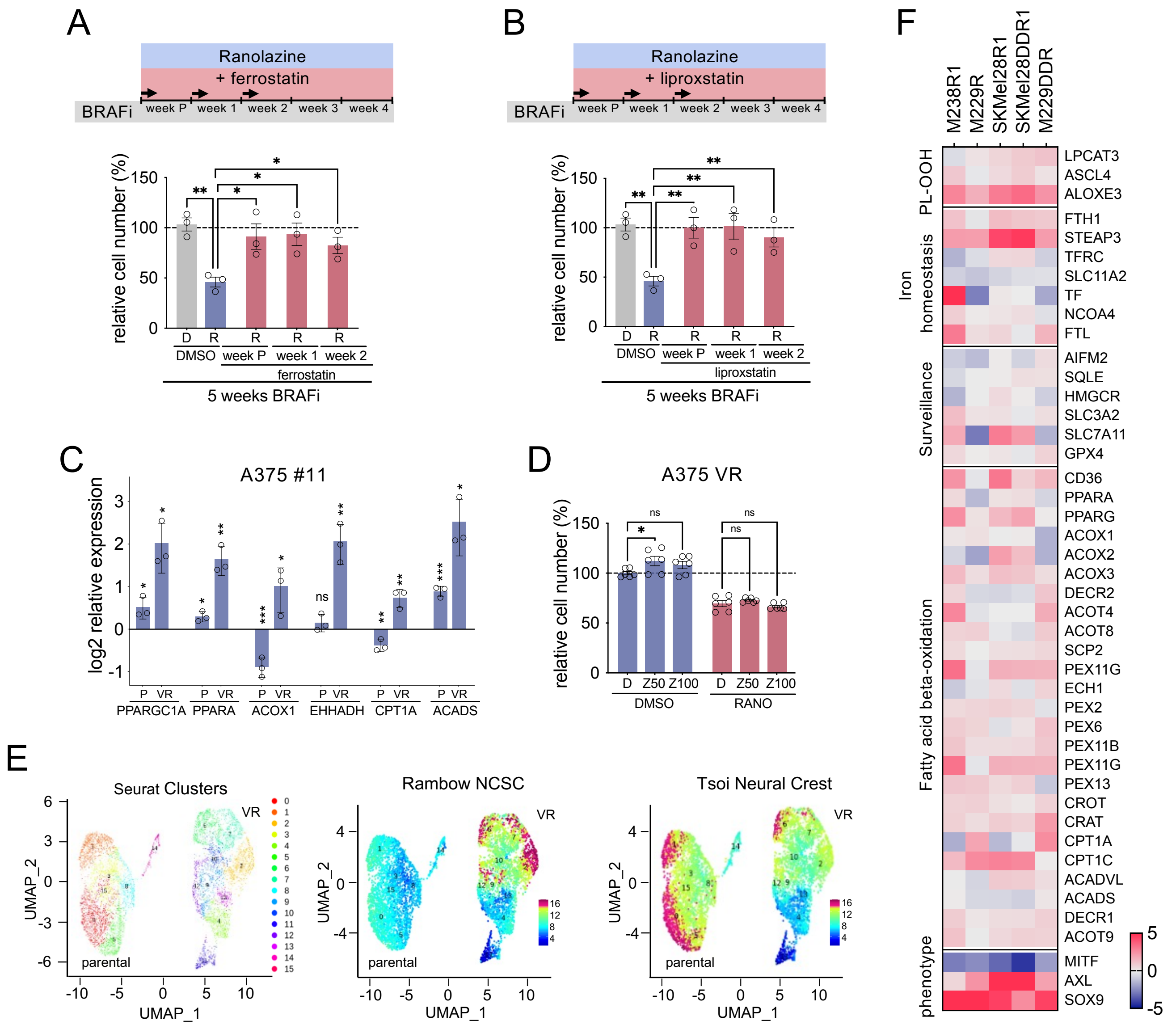

Figure S1: RANO promotes ferroptosis in melanoma cells during BRAFi resistance acquisition

A, B Colony formation assay (CFA) quantification for A375 cells treated with vemurafenib (BRAFi) and ranolazine (R) for 5 weeks (1 week (week P), 5µM vemurafenib, followed by 4 weeks 0,5 µM vemurafenib). The protocol for monitoring the establishment of vemurafenib persisters (P) in week P and resistance during week 1-4 is shown. (**A**) Ferrostatin-1 or (**B**) liproxstatin-1 was added at the beginning of the indicated weeks. The cell number of BRAFi treated cells with addition of DMSO was set 100%. (*n* = 3, Mean ± SEM, Holm-Sidák test of one-way ANOVA. *p-value ≤ 0.05; **p-value ≤ 0.01). **C** RT-qPCR analysis of the indicated genes in persister (P) and resistant (VR) cells compared to parental A375 cells. (*n* = 3, Mean ± SD, two-tailed unpaired *t*-test. *p-value ≤ 0.05; **p-value ≤ 0.01). **D** CFA quantification of A375VR cells treated with DMSO or ranolazine (RANO) in presence of 50 µM or 100 µM Z-DEVD-FMK (Z) or DMSO (D). (*n* = 6, Mean ± SEM, Holm-Sidák test of one-way ANOVA. *p-value ≤ 0.05). **E** UMAP visualization of 3,676 parental and 3,777 VR cells coloured by Seurat clusters and by the expression of the Rambow NCSC (32) and Tsoi Nerual Crest (5) state. **F** Heatmap of the log2FC in the expression of the indicated genes in BRAFi resistant (R) or BRAFi/MEKi double-resistant (DDR) melanoma cells compared to the respective parental cell lines taken from GSE65185.

Redondo-Muñoz et al, Supplementary Figure 2
(corresponding to Figure 4)

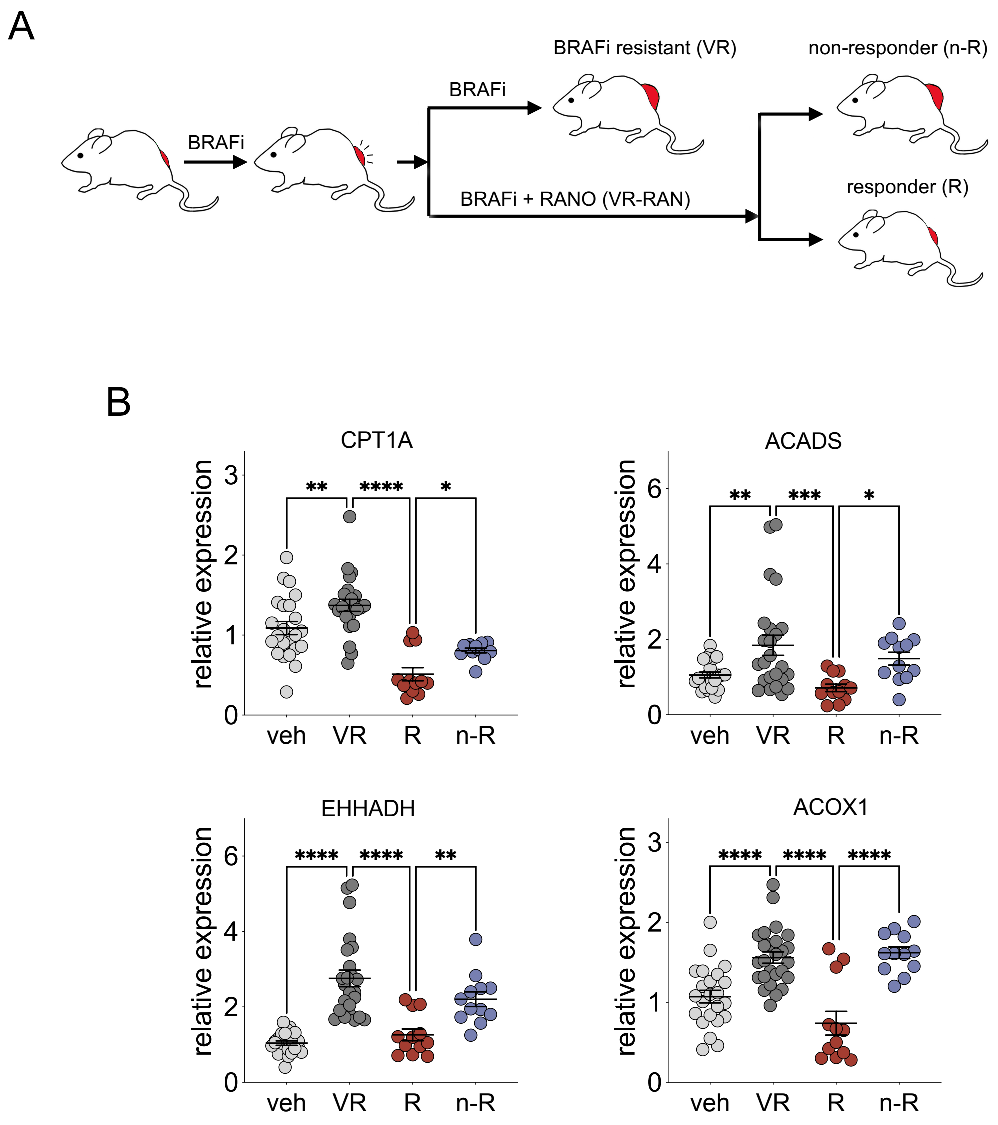

Figure S2: Expression of FAO regulators in BRAFi-RANO resistant tumours

A Schematic of the treatment protocol as previously described (15) and sample assignment. **B** RT-qPCR analysis of the indicated genes in A375 tumours from mice treated as indicated. Data are triplicates from *n* = 8 tumours for vehicle (veh) or BRAFi (VR), and *n* = 4 tumours for RANO responder (R) or RANO progressed (n-R). (Mean ± SEM, uncorrected Fisher's LSD test of one-way ANOVA. *p-value ≤ 0.05; **p-value ≤ 0.01; ***p-value ≤ 0.001; ****p-value ≤ 0.0001).

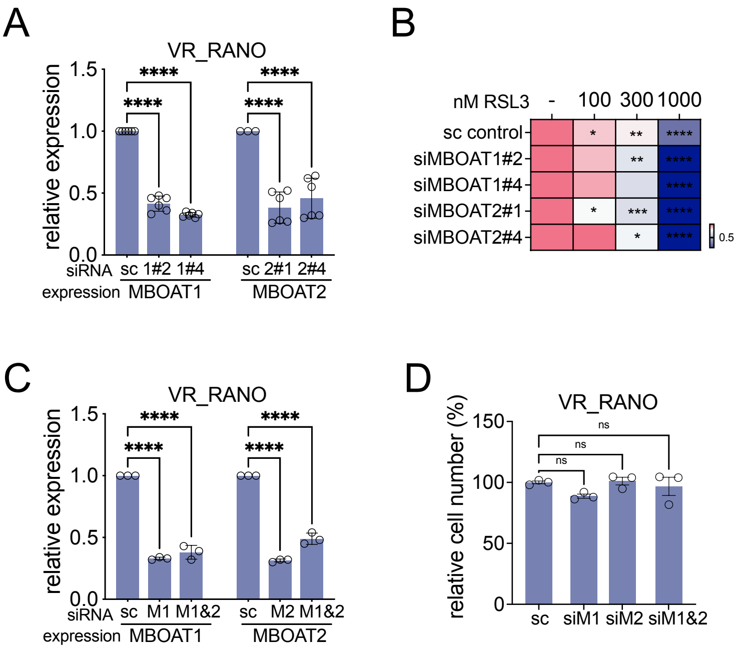

Figure S3: MBOAT1 and MBOAT2 expression affects RSL3 sensitivity

A RT-qPCR analysis of MBOAT1 and MBOAT2 expression in VR-RANO cells after treatment with the indicated siRNAs. (*n* = 6, Mean ± SD, Sidák test of one-way ANOVA. ****p-value ≤ 0.0001). **B** **C** RT-qPCR analysis of MBOAT1 and MBOAT2 expression in VR-RANO cells after treatment with the indicated siRNAs. (*n* = 3, Mean ± SD, Sidák test of one-way ANOVA. ****p-value ≤ 0.0001). **D** CFA quantification of VR-RANO cells transfected with a control (sc) siRNA or siRNAs targeting MBOAT1 or MBOAT2 alone or in combination. (*n* = 3, Mean ± SEM, Holm-Sidák test of one-way ANOVA).

Redondo-Muñoz et al, Supplementary Figure 4
(Figure 7)

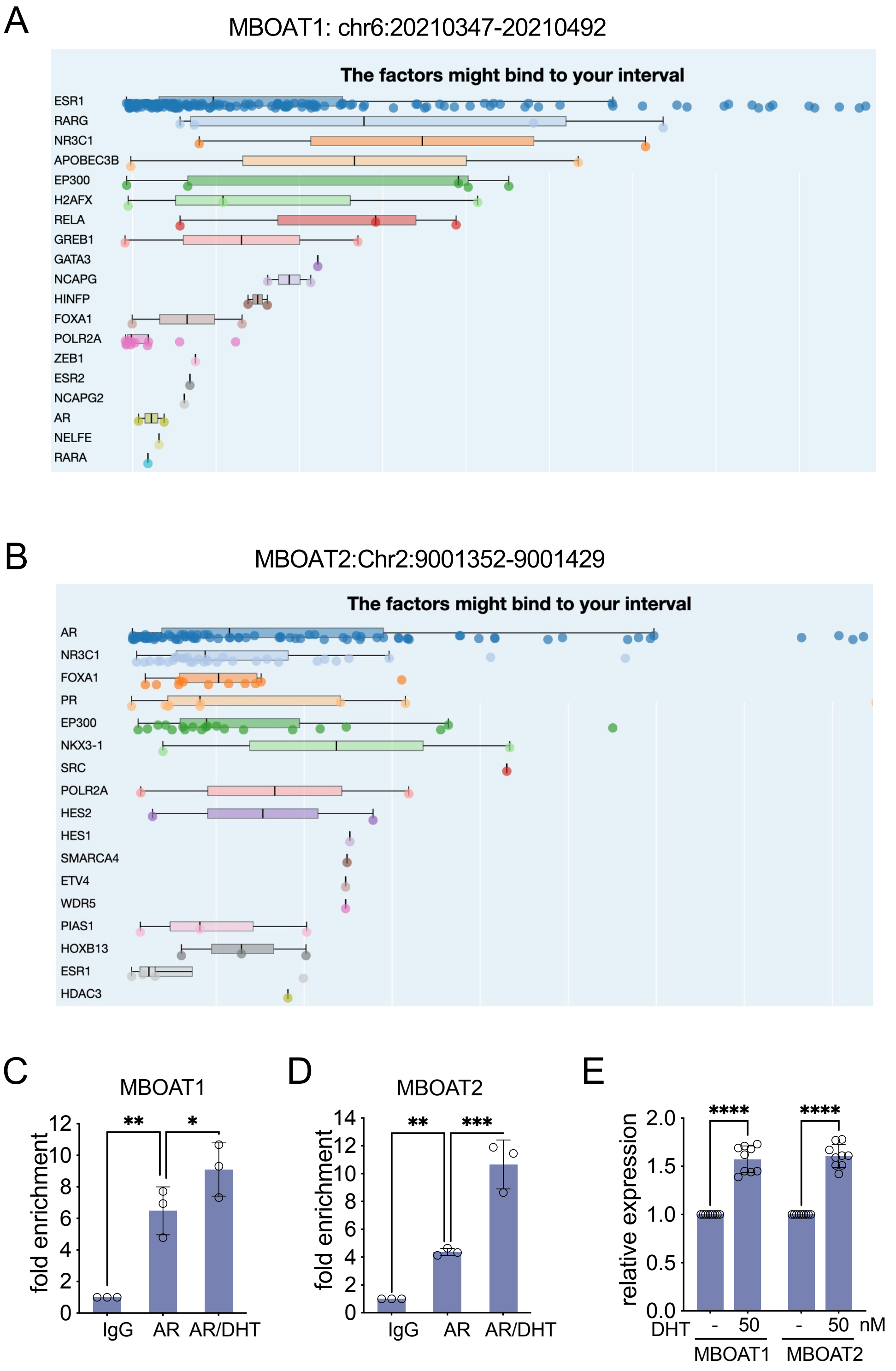

Figure S4: AR regulates MBOAT1/2 expression in resistant melanoma cells

A, B Interrogation of publicly available ChIP-seq data of indicated chromosome regions corresponding to **A**, *MBOAT1* and **B**, *MBOAT2* for putative ARE sites using http://dbtoolkit.cistrome.org. (38, 39). **C, D** ChIP-qPCR showing the occupancy of AR on the (**C)** human *MBOAT1* and (**D)** human *MBOAT2* intervals indicated in (**A)** and (**B),** respectively in VR_RANO cells untreated or treated with 50 nM DHT for 24 h. (*n* = 3, Mean ± SD, Holm-Sidák test of one-way ANOVA. *p-value ≤ 0.05; **p-value ≤ 0.01; ***p-value ≤ 0.001). **E** RT-qPCR for MBOAT1 and MBOAT2 in VR_RANO cells treated 50 nM DHT for 24 h. (*n* =9, Mean ± SD, two-tailed unpaired t-test. ****p-value ≤ 0.0001).

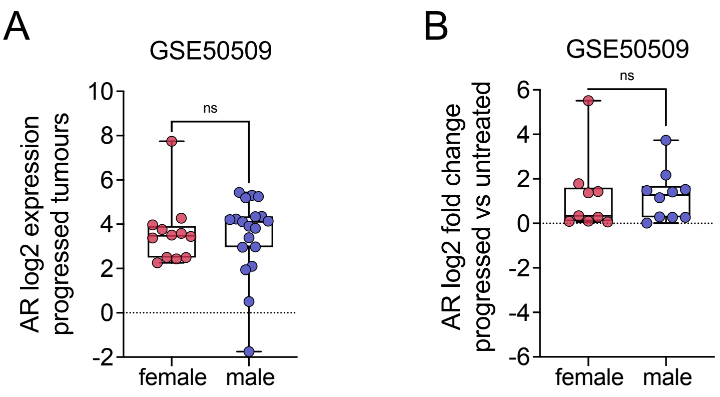

C

GSE50509	
Patient	Sex
2	F
8	F
9	F
10	F
11	F
21	F
23	F
1	M
3	M
4	M
5	M
7	M
12	M
13	M
14	M
17	M
18	M
24	M
25	M
28	M
30	M

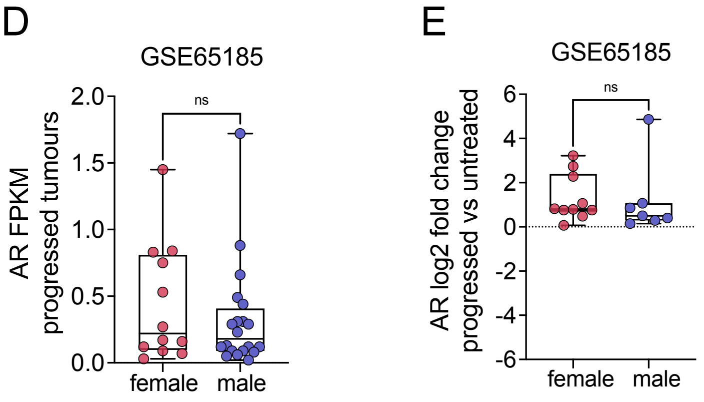

F

GSE65185	
Patient	Sex
3	F
8	F
9	F
15	F
17	F
1	M
2	M
4	M
5	M
6	M
10	M
16	M
20	M

Figure S5: AR expression in BRAFi progressed tumours does not differ between male and female patients

A, Comparative analysis of the basal expression of AR in progressed tumours in the GSE50509 cohort. (*n* = 12 female; *n* = 19 male; Boxplots reflect lower quartile, median, and upper quartile. Whiskers indicate min to max, two-tailed Welch's test). **B,** Fold change in AR expression in the 'AR-high' progressed compared to untreated tumours for the indicated sex of the individual patients in the GSE50509 cohort. (*n* = 9 female; *n* = 10 male; Boxplots reflect lower quartile, median, and upper quartile. Whiskers indicate min to max, two-tailed Welch's test). **C**, Patient information in the GSE50509 cohort. **D,** Comparative analysis of the basal expression of AR in progressed tumours in the GSE65185 cohort. (*n* = 12 female; *n* = 20 male; Boxplots reflect lower quartile, median, and upper quartile. Whiskers indicate min to max, two-tailed Welch's test). **E,** Fold change in AR expression in the 'AR-high' progressed compared to untreated tumours for the indicated sex of the individual patients in the GSE65185 cohort. (*n* = 10 female; *n* = 7 male; Boxplots reflect lower quartile, median, and upper quartile. Whiskers indicate min to max, two-tailed Welch's test). **F**, Patient information in the GSE65185 cohort.

Redondo-Muñoz et al, Supplementary Figure 6
(Figure 8)

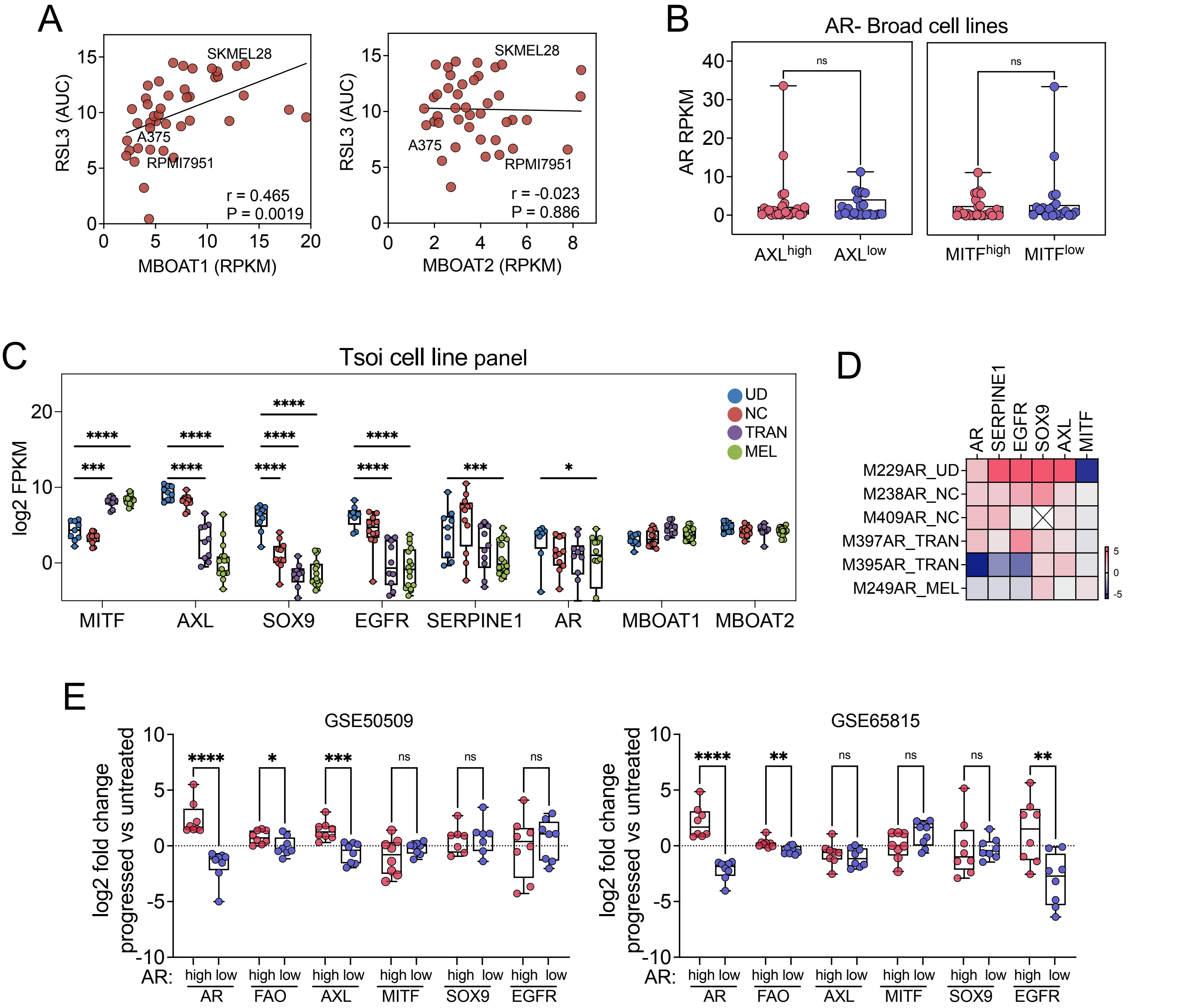

Figure S5: Correlation of AR and MBOAT1/2 expression with melanoma phenotype

A Pearson correlation analysis of MBOT1 or MBOAT2 expression (FPKM, fragments per kilobase of exon per million fragments) versus RSL3 sensitivity (area under curve; AUC) as obtained from the Cancer Therapeutics Response Portal (CTRP); http://portals.broadinstitute.org/ctrp/. **B** Correlation analysis of AR expression with high or low AXL or MITF expression in the Broad melanoma cell line panel. (Boxplots, whiskers indicate min to max. Two-tailed unpaired t-test). **C** Analysis of GSE80829 for the expression of the indicated genes in the Tsoi panel of naïve melanoma cells lines: UD, undifferentiated; NC, neural crest-like; TRAN, transitory; MEL, melanocytic. (UD, *n* = 9; NC, *n* = 12, TRAN, *n* = 10, MEL, *n* = 16. Boxplots reflect lower quartile, median, and upper quartile. Whiskers indicate min to max. Tukey test of 2-way ANOVA. *p-value ≤ 0.05; ***p-value ≤ 0.001; ****p-value ≤ 0.0001). **D** Heatmap of the log2FC in the expression of the indicated genes in the indicated Tsoi (5) BRAFi resistant (R) melanoma cells compared to the respective parental cell lines. **E** Comparative analysis of the log2 FC of AR, FAO regulators and the indicated UD state markers in tumour datasets GSE50509 and GSE65185 stratified for high or low AR expression. (Upper and lower quartile, *n* =8, one-way ANOVA with unpaired t-test with Welsh correction. *p-value ≤ 0.05; **p-value ≤ 0.01; ***p-value ≤ 0.001; ****p-value ≤ 0.0001).
